# Poster Session II A322 BACTERIA-DERIVED SATURATED AND MONOUNSATURATED LYSOPHOSPHOLIPIDS CONTRIBUTE TO VISCERAL PAIN IN PATIENTS WITH IBS

**DOI:** 10.1093/jcag/gwaf042.322

**Published:** 2026-02-13

**Authors:** M Quaderi, J Pujo, F A Vicentini, G De Palma, S Collins, P Bercik

**Affiliations:** Biochemistry & Biomedical Sciences, McMaster University Faculty of Health Sciences, Hamilton, ON, Canada; Gastroenterology, Farncombe Family Digestive Health Research Institute, Hamilton, ON, Canada; McMaster University, Hamilton, ON, Canada; McMaster University, Hamilton, ON, Canada; Gastroenterology, Farncombe Family Digestive Health Research Institute, Hamilton, ON, Canada; Medicine, McMaster University, Hamilton, ON, Canada

## Abstract

**Background:**

Irritable bowel syndrome (IBS) is a disorder of gut–brain interaction characterized by abdominal pain and altered bowel habits, affecting up to 10% of individuals worldwide. IBS has a significant impact on patients’ mental health, reducing their overall quality of life. Pathophysiology of visceral hypersensitivity, a key driver of abdominal pain in IBS, is poorly understood but accumulating evidence suggests that microbial metabolites can trigger visceral nociception. Lysophosphatidylcholine (LPC) and its metabolite lysophosphatidic acid (LPA) are a group of bioactive phospholipids previously linked to the development of neuropathic pain. However, the specific species of LPC and LPA produced by gut microbiota that are involved in genesis of abdominal pain in IBS remain unknown.

**Aims:**

The purpose of this study is to identify altered LPC and LPA species in fecal samples from patients with IBS compared to healthy individuals, and to assess their ability to stimulate nociceptive neurons.

**Methods:**

Fecal samples were collected from 27 IBS patients during periods of minimal/no abdominal pain and severe abdominal pain, as well as from 17 healthy controls. Phospholipids were extracted from fecal samples using Bligh-Dyer and solid phase extraction techniques and analyzed using targeted and untargeted mass spectrometry. In targeted approach, the fecal concentrations of selected phospholipid species including LPC 16:0, 18:0, 18:1, 18:2, 20:0, 22:0, 24:0 and LPA 16:0, 18:0, 18:1, 18:2, 20:0, 20:4 were measured. Calcium mobilization in response to LPC 16:0 and LPA 18:1 treatment (at 1 μM and 10 μM) was assessed in dorsal root ganglia (DRG) sensory neurons of C57BL/6 mice using a calcium imaging reader (Cytation C10) with the fluorescent probe Fluo-4 (1mM).

**Results:**

Untargeted metabolomic analysis has revealed altered levels of multiple phospholipid species, including LPC 16:0, 18:0, 22:4 and LPA 18:1, in patients with IBS compared to healthy controls (all p < 0.05). Further analysis of IBS samples using targeted mass spectrometry demonstrated increased levels of LPC 16:0, LPA 18:0, and LPA 18:1 during episodes of severe abdominal pain compared to episodes of minimal/no abdominal pain (p < 0.01, p = 0.04, p = 0.03, respectively). Compared with vehicle (PBS), the fraction of DRG neurons responding to LPC 16:0 and LPA 18:1 treatment (both at 10 μM) was significantly higher (p < 0.01 and p = 0.03, respectively).

**Conclusions:**

Several saturated and monounsaturated LPC and LPA species are altered during episodes of severe abdominal pain in IBS patients, some of them displaying nociceptive properties *in vitro*. Further studies are needed to investigate the pain modulatory properties of the remaining species.

**Funding Agencies:**

CIHR

